# Clinical and biological features of complicated and asymptomatic malaria among PLHIV and HIV-negative adults: An observational study in Libreville, Gabon

**DOI:** 10.1371/journal.pone.0327068

**Published:** 2026-02-17

**Authors:** Bridy Chesly Moutombi Ditombi, Charleine Manomba Boulingui, Ornella Anaïse Mbang Nguema, Coella Joyce Mihindou, Bedrich Pongui Ngondza, Jacques Mari Ndong Ngomo, Magalie Essomeyo Mebale, Michèle-Marion Ntsame Owono, Ardin Dimitri Mabicka Moussavou, Christian Mayandza, Noé Patrick Mbondoukwé, Helena Noeline Kono, Marielle Karine Bouyou Akotet

**Affiliations:** 1 Department of Parasitology-Mycology and Tropical Medicine, Université des Sciences de la Santé, Libreville, Gabon; 2 Infectiology Diseases Ward, Centre Hospitalier Universitaire de Libreville, Gabon; Bernhard-Nocht-Institute for Tropical Medicine (BNITM), GERMANY

## Abstract

Malaria and HIV remain major public health challenges in sub-Saharan Africa. This study described the spectrum of malaria in people living with HIV (PLHIV) and HIV-negative adults in Gabon, where both diseases are endemic.

**Methods:**

A cross-sectional study was conducted at the Centre Hospitalier Universitaire de Libreville from September 2021 to October 2022. Asymptomatic and febrile PLHIV and HIV negative volunteers were screened for malaria and *Plasmodium*-infected individuals enrolled in the study. Data collected included socio-demographic characteristics, clinical presentation, cotrimoxazole use, antiretroviral treatment, and laboratory parameters, such as parasitaemia, haemoglobin levels and CD4^+^ cell counts. Data from PLHIV and HIV-negative participants were compared using bivariate and multivariable logistic regression analysis.

**Results:**

Among the 1.192 individuals tested, 513 were PLHIV and 599 HIV-negative. Overall, 56 *Plasmodium falciparum*-infected PLHIV and 66 HIV-negative individuals were enrolled. Asymptomatic malaria was more frequent among HIV-negative participants (60.6% *vs* 25.0%), while severe malaria was significantly more common among HIV-positive patients (48.2% *vs* 12.1%) (p < 0.01). Prostration (16.7%), repeated seizures (14.3%), impaired consciousness (26.2%), severe anaemia (14.3%) and hypoglycemia (11.9%) predominated in PLHIV. Conversely, vomiting (26.9%) and high parasitemia (23.1%) were more frequent in HIV-negative patients. CTX chemoprophylaxis was associated with a reduction in *Plasmodium falciparum* parasitemia (*p = 0.03*). The median CD4 + cell count was significantly lower in PLHIV with severe malaria (150 [IQR: 90-204] cells/µL) compared to those with asymptomatic (304 [IQR: 189-476] cells/µL) (*p = 0.018*).

**Conclusion:**

Compared to HIV negative, asymptomatic malaria was less frequent while severe malaria predominantly affects PLHIV with advanced immunosuppression, highlighting the importance of early detection and management of co-infection to reduce morbidity. Further studies are needed to investigate the neurological manifestations and the impact of prophylactic strategies in Libreville.

## Introduction

Malaria remains a major public health problem worldwide, with WHO estimating that there were 263 million malaria cases and 597000 deaths worldwide in 2023 [1]. Despite numerous efforts, sub-Saharan Africa (SSA) remains the most affected region. Human immunodeficiency virus (HIV) is also a significant public health concern; in 2023, 39.9 million people were living with HIV, with Africa accounting for the highest number of new infections (540 000) and deaths (390 000) [2].

In SSA, malaria and HIV/AIDS often co-occur in vulnerable populations, posing a synergistic health challenge [3]. Several studies have shown that adults living with HIV are at increased risk of developing severe malaria, particularly cerebral malaria and severe anaemia [4,5]. Indeed, HIV infection may increase the risk of complicated and severe malaria and death, as well as malaria-related mortality in both children and adults [6]. This interaction may occur because HIV infection causes cellular immunosuppression potentially impairing the immune response to malaria [7]. Furthermore, HIV is associated with a reduced ability of people living with HIV (PLHIV) to either prevent or suppress malaria parasitemia, leading to a higher incidence of clinical disease [8,9].

Gabon is endemic for both diseases, with stable malaria transmission throughout the year, and a prevalence reaching over 40% [10]. HIV prevalence was estimated at 3.6% in the last demographic and health survey [11]. Few studies have explored the prevalence of malaria among adult PLHIV in Gabon [12,13]. Existing data suggest that CTX, which is recommended in national guidelines for the prevention of opportunistic infections, is associated with reduced malaria parasitemia [13,14]. However, the clinical spectrum of malaria in HIV co-infected individuals remains under-characterized in the country, particularly the prevalence of neurological symptoms and severe disease which are associated with poor outcomes. Recent observations suggest a shift towards neurological presentations, especially in *Plasmodium (P.) falciparum*-infected older children and young adults [10].

In routine clinical practice, because toxoplasmosis and tuberculosis are the main causes of hospitalization for febrile illness among adult PLHIV in Gabon, malaria is less frequently suspected due to its non-specific symptoms [15,16]. Malaria tends to present with greater severity in PLHIV, underscoring the need for improved clinical recognition and timely management. This study therefore aimed to describe and compare the clinical and biological characteristics of malaria including asymptomatic, moderate, and severe forms among HIV-positive and HIV-negative adults in an urban endemic setting.

## Methodology

### Study design and setting

This cross-sectional study was conducted from September 2021 to October 2022 in the Infectious Diseases Ward of the Centre Hospitalier Universitaire de Libreville (IDW-CHUL), a national referral hospital for infectious disease care in Gabon. During the study period, this ward was the only referral center for the management of HIV infection in Gabon, and especially in Libreville were the majority of PLHIV are managed. The study was implemented across three units of the IDW-CHUL: the inpatient unit where febrile PLHIV and HIV negative patients are usually hospitalized for suspected infectious diseases, including malaria; the outpatient HIV care clinic where asymptomatic PLHIV attend their routine follow-up visit and; the general outpatient unit where apparently healthy adults participating in a screening for a survey on intestinal parasite carriage during the same period were approached.

### Study population and recruitment procedures

Participants were consecutively enrolled from the inpatient and outpatient units. Each morning, a brief sensitization session was conducted in both units by trained staff to inform patients and caregivers about the study objectives. Potential participants were approached individually after verbal consent was obtained for eligibility screening.

The screening was performed for those aged ≥ 18 years, who had a negative HIV test or where known HIV positive, and who underwent malaria diagnosis using expert microscopy. Only individuals with *Plasmodium sp* parasitaemia confirmed by microscopy positive blood smear (PBS) were eligible for the study analysis. Based on their clinical presentation and recruitment setting, individuals were stratified into four groups (symptomatic PLHIV, symptomatic HIV-negatives, asymptomatic PLHIV and asymptomatic HIV-negatives) according to the following inclusion criteria.

For symptomatic PLHIV and HIV-negative participants, criteria were a current fever (axillary temperature ≥ 37.5°C) or recent history of fever (72 hours); a confirmed *Plasmodium* infection by microscopy and no other identified cause of febrile illness after routine diagnosis (full clinical and routine laboratory testing).

For asymptomatic PLHIV, inclusion criteria were: attending their scheduled six-monthly routine HIV care follow-up at IDW-CHUL, being apparently asymptomatic without current or recent history of fever or malaria-like symptoms for at least 28 days; confirmed *Plasmodium* parasitaemia.

Asymptomatic HIV-negative participants were identified from a group of apparently healthy volunteers not enrolled in the PARCAM study which required infection free participants, who had confirmed HIV-negative status based on negative HIV test results, negative screening for other infectious diseases (Hepatitis B, Hepatitis C, syphilis…), no current symptoms suggestive of acute infection or any other illness, microscopy-confirmed asymptomatic *Plasmodium* infection [17].

All participants signed informed consent.

The exclusion criteria were the same for all participants: pregnancy, history of antimalarial and antibiotic treatment within the past 28 days, presence of any major opportunistic infection or acute medical condition including COVID-19 requiring emergency care (e.g., tuberculosis, cryptococcosis, toxoplasmosis), incomplete diagnostic or demographic data and refusal to participate or consent withdrawal.

### Sample size calculation

As prior data on clinical malaria in adults were lacking in Gabon, the minimum number of participants to be screened for malaria was estimated based on the prevalence of asymptomatic malaria among adults, assuming similar recruitment targets for febrile patients to obtain balanced group sizes. The sample-size estimation was therefore performed according to HIV status rather than fever status, as the primary aim was to assess the characteristics of symptomatic malaria and asymptomatic *P.falciparum* carriage in PLHIV and HIV negative.

Considering the reported prevalence of asymptomatic malaria in PLHIV (7.1%) and HIV-negative individuals (13%), the minimum sample size was calculated using the formula for comparing two independent proportions with a required power of 80% and a 95% confidence level, using a precision level of 5% [12,18].


$$n=\frac{{(Z}_{\alpha /\text{2}}\sqrt{\text{2}p_{\text{1}}(\text{1}-p_{\text{1}}})+Z_{\beta }\sqrt{p_{\text{1}}(\text{1}-p_{\text{1}})+p_{\text{2}}(\text{1}-p_{\text{2}}){)}^{\text{2}}}}{|\;p_{\text{2}}-p_{\text{1}}{|}^{\text{2}}\;}$$

Assuming a prevalence of 7.1% among HIV-positive individuals (P₁ = 0.071) and 13% among HIV-negative individuals (P₂ = 0.13), with a significance level of 0.05 (95% confidence) and a power of 80% (1 – β = 0.80), the pooled prevalence was calculated as P = (P₁ + P₂)/2, and the corresponding Z-values were Z₁–α/2 = 1.96 and Z₁–β = 0.84 for sample size estimation.

This led to a target of a minimum of 407 PLHIV and 407 HIV negative in dividuals to be screened. All consenting participants were prospectively included using a non-convenient sampling approach during the study period.

### Data collection

Clinical and biological data of study participants were recorded using a standardised case report form, which was developed and validated prior to study initiation. The form included sociodemographic information (age, gender), body temperature, clinical signs, and laboratory parameters (parasitaemia, haemoglobin (Hb) level, blood glucose, liver function tests, creatinine, and CD4^+^ cell count), data on cotrimoxazole (CTX) chemoprophylaxis and antiretroviral treatment (ART) were also collected.

### Malaria diagnosis

Thick smears were prepared according to the Lambaréné method described by Planche et *al* [19]. Briefly, 10 μL of blood was collected using a pipette and placed as a drop on the lower third of a microscope slide. The blood was then spread over a rectangular area of 180 mm^2^ [19]. The slide was air-dried at room temperature and stained for 15 minutes with a 10% Giemsa solution freshly prepared with buffer at pH 7.2. It was then rinsed with clean water and air dried at laboratory temperature.

In addition, a thin smear was prepared on the same slide for *Plasmodium* species identification. A 5 μL drop of blood was placed on a slide and spread along the edge by capillarity using a second slide. The smear was then fixed with absolute methanol and allowed to dry. It was subsequently stained with 10% Giemsa solution for 15 minutes, rinsed with clean water, and air dried at laboratory temperature.

Each smear was examined for asexual forms of *Plasmodium spp* detection using light microscope under oil immersion. Malaria diagnosis was based exclusively on the presence of asexual blood-stage forms of *Plasmodium sp* detected by microscopy. Parasite density was expressed as the number of asexual parasites per uL (p/uL). A double-reading protocol was implemented to ensure the accuracy and reliability of results. In cases of discordant findings between the two primary readers (defined as disagreement in parasite detection or a significant discrepancy in parasitaemia quantification), the slide was re-examined by a third independent experienced reader. The mean of the two closest parasitaemia values was used for analysis. Slides were considered negative if no *Plasmodium* parasites were observed after examination of 100 high-power fields. High parasitaemia was defined as a parasite density greater than 100.000 p/µL according to cut-off previously used infected adults from Libreville and other cities [20]. This cut-off allows comparison across HIV groups receiving cotrimoxazole prophylaxis.

### Haematological and biochemistry parameters

Haemoglobin (Hb) levels were measured using a Sysmex XN350 analyser. In this study, anaemia was defined as a Hb concentration below 11 g/dL, following the national laboratory standards and thresholds commonly used in malaria and HIV research in Gabon [13,21]. Thus, anaemia was categorised according to Hb level in mild anaemia (Hb 10–9.0 g/dL), moderate anaemia (Hb 8.9–5.0 g/dL), and severe anaemia (Hb < 5.0 g/dL). Biochemical tests were performed to assess liver function, creatinine, and blood glucose levels using the Pentra C200 biochemistry analyser. Liver cytolysis was defined as a aspartate aminotransferase (AST) or alanine aminotransferase (ALT) levels exceeding three times the upper limit of normal values. Hypoglycaemia was defined as a blood glucose concentration ≤ 2.2 mmol/L.

### Definitions of malaria cases

Asymptomatic malaria was defined as the presence of malaria parasites in peripheral blood (parasitemia) in individuals who had not received an antimalarial treatment within the previous 28 days and who had neither experienced a fever, nor shown clinical signs suggestive of malaria.

For febrile participants, malaria was classified as complicated or clinical malaria only when no alternative cause of fever was identified after a full clinical evaluation and complementary investigations (haematology, urinalysis, chest radiography, and targeted microbiological tests). According to WHO guidelines, severe malaria was defined by the presence of one or more of the following neurological symptoms: impaired consciousness (including unresponsiveness or coma); prostration, i.e., generalized weakness such that the patient is unable to sit, stand or walk without assistance; repeated convulsions (more than two episodes in 24 hours); deep breathing and acute respiratory distress syndrome; acute renal failure; acute pulmonary oedema; circulatory collapse or shock; clinical jaundice with evidence of other vital organ dysfunction; haemorrhagic abnormalities, severe anaemia (Hb < 5 g/dL), spontaneous bleeding, hypoglycaemia (glycaemia < 2.2 mmol/L) [22].

Moderate malaria was defined as the presence of *Plasmodium* parasitemia in hospitalized patients who did present any WHO criteria for severe malaria.

Thus, complicated malaria included both moderate or severe malaria hospitalized cases.

### Ethics approval and consent to participate

The study was approved by the CS-COVID-19 who provide approval for all research activities during the acute the health emergency period related to the COVID-19 epidemic phase (0015/P/COPIL/CSCOVID-19) and the Gabonese National Research Ethics Committee (PROT N°027/022/CNE/P) and was part of the CANTAM3-EPI project. Written informed consent was obtained from all participants or, in case of unconsciousness, from a legal representative. The study was conducted according to the principles expressed in the Declaration of Helsinki. All infected participants were treated according to national guidelines. When neurological signs were present, alternative diagnoses such as toxoplasmosis and neuro-meningeal cryptococcosis were systematically excluded.

### Statistical analysis

Data were analysed using R version X.X. Continuous variables were tested for normality using the Shapiro-Wilk test. Because most variables were non-normally distributed, results are expressed as median [interquartile range] and compared using the Mann-Whitney U or Kruskal-Wallis tests. Categorical variables were compared using the χ² test or Fisher’s exact test, as appropriate. Variables with a *p*-value < 0.20 in univariate analysis were entered into the multivariable model, and results were expressed as adjusted odds ratios (aOR) with 95% confidence intervals. A multivariable logistic regression models was performed to identify factors associated with clinical malaria. Independent variables included age (years), sex, HIV status, CD4 ⁺ count categories and cotrimoxazole prophylaxis. Model fit was assessed using the Hosmer-Lemeshow goodness-of-fit test, and multicollinearity was checked using the variance inflation factor (VIF). *P*-values below 0.05 indicate statistically significant associations. Clinical comparisons, including malaria type and severity, were performed exclusively among symptomatic participants, who were all recruited under comparable hospital-based conditions. Asymptomatic participants, identified through routine screening in outpatient settings, were included only for parasitological comparisons (parasite carriage and density).

## Results

**Fig 1:**
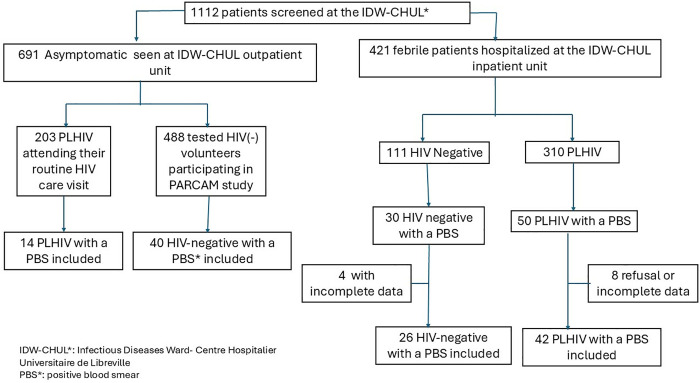
Flow diagram of participant recruitment and inclusion.

A total of 1112 adults were screened for malaria; 513 were PLHIV and 599 were HIV-negative, 122 individuals with confirmed *Plasmodium falciparum* infection (56 PLHIV and 66 HIV-negative) were finally included for the comparative analysis of malaria related features (Fig 1).

The median age did not differ significantly between PLHIV and HIV-negative individuals (*p = 0.75*) (Table 1). The sex ratio was towards females, with nearly two-thirds of the study population being female; among the 44 men 16 were PLHIV and 28 HIV negative.

**Table 1:** General characteristics of study population.

Variables	Total N = 122	PLHIV N = 56	HIV-negative N = 66	*p*
**Age in years [IQR],**	39.0 [30-40]	39.5 [32-46]	38.5 [25-52]	*0.75*
**Female, n (%)**	78(63.9)	40 (71.4)	38(57.6)	*0.11*
**Temperature [IQR],** (**°C**)	38.5 [38-39]	38.1 [37.2-38.8]	39.0 [38-39.8]	*<0.01*
**Type of malaria, n (%)**				*<0.01*
Asymptomatic	54 (44.3)	14 (25.0)	40 (60.6)	
Moderate	33 (27.0)	15 (26.8)	18 (27.3)	
Severe	35 (28.7)	27 (48.2)	8 (12.1)	
Clinical form*, n (%)				
Neurological	28 (22.9)	23 (54.8)	3 (11.5)	*0.02*
Respiratory	4 (3.3)	2 (4.7)	2 (7.7)	*0.6*
Digestive	9 (7.4)	8 (19.0)	1 (3.8)	*0.05*
**Parasitemia [IQR],** (p/µl)	166 [25-1027]	280 [97-813]	60 [10-2500]	*0.02*
**Hb median [IQR],** (g/dL)	10.2 [8.3-12.1]	9.1 [7.5-10.7]	11.9 [10.1-13.3]	*<0.01*
Anemia severity**, n (%)				*<0.01*
Mild	27 (27.0)	17 (30.3)	10 (22.7)	
Moderate	23 (23.0)	19 (33.9)	4 (9.1)	
Severe	7 (7.0)	6 (10.7)	1 (2.3)	
**Creatinine [IQR]**, (ml/min)	98.0 [57.0-152.6]	98.0 [57.8-154,2]	103.3[56.3-147.7]	*0.89*
**AST [IQR],** (UI/mL)	32.0 [19.1-51.5]	37.0 [23.1-57.0]	21.8 [14.5-32.0]	*<0.01*
**ALT [IQR],** (UI/mL)	29.0 [16.0-50.7]	38.0 [20.6-53.0]	18.0 [12.8-45.2]	*0.03*

*One patient could have more than one form

**Number of patients who have haemoglobin level available (N = 100)

According to the pre-sceening result, the prevalence of complicated malaria was 16.1% (n = 50/310) among febrile PLHIV and 27.0% (n = 30/111) among febrile HIV-negative participants (see Fig 1). The prevalence of asymptomatic malaria was 8.2% (n = 40/488) in HIV-negative participants and 6.9% (n = 14/203) in PLHIV. *Plasmodium (P.) falciparum* was the only species identified. Severe malaria was significantly more common among PLHIV (*p < 0.01*). Neurological symptoms were the most frequently reported clinical manifestations (*p < 0.02*) (Table 1).

Overall, the median parasitemia was significantly higher in PLHIV compared to HIV-negative individuals (*p = 0.02*). Hb levels were measured for 100 participants and 57 (57.0%) had a level below 11g/dL. Median Hb concentrations were significantly lower in PLHIV than in the HIV-negative individuals (*p* < 0.01), with 75.0% of HIV-positive patients presenting with anaemia compared to 22.7% of HIV-negative patients (*p* < 0.01). Elevated liver function test (LFT) values were also more often observed in HIV-positive patients (Table 1). The median CD4^+^cell count among PLHIV was 190 [111–311] cells/mm^3^.

### Clinical characteristics and blood parameters of malaria according to HIV group

Table 2 presents the clinical characteristics and blood parameters of febrile patients with complicated malaria, stratified by HIV status. The incidence of asthenia and prostration was recorded, with higher rates observed in HIV-negative patients, though these differences were not statistically significant. A trend towards increased neurological signs was noted in PLHIV, with these patients appearing to be at higher risk of repeated seizures and impaired consciousness (Table 2). Vomiting occurred significantly more frequently in the HIV-negative group (p = 0.02).

**Table 2:** Clinical and biological features associated with HIV-malaria coinfection.

	n	%	Adjusted OR [CI95%]	*p*
**Asthenia**				
PLHIV	2	4.8	0.13 [0.09-1.4]	*0.27*
HIV negative	5	19.21	Ref	
**Prostration**				
PLVIH	7	16.7	1.3[0.4-2.1]	*0.4*
HIV negative	2	7.7	Ref	
**Repeated seizures**				
PLVIH	6	14.3	5.0 [0.9-7.4]	*0.07*
HIV negative	1	3.9	Ref	
**Impaired consciousness**				
PLHIV	11	26.2	NA	*0.06*
HIV negative	0.0	0.0	NA	
**Coma**				
PLHIV	2	4.8	0.47 [0.02-1.9]	*0.22*
HIV negative	3	11.5	Ref	
**Vomiting**				
PLHIV	1	2.4	0.11 [0.02-0.29]	*0.02*
HIV negative	7	26.9	Ref	
**Diarrhea**				
PLHIV	8	19.0	NA	*0.11*
HIV negative	0	0.0	NA	
**Respiratory distress**				
PLHIV	2	4.8	0.4 [0.05-2.1]	*0.69*
HIV negative	2	7.7	Ref	
**Acute renal failure**				
PLHIV	16	38.1	1.2 [0.4-1.8]	*0.17*
HIV negative	8	30.8	Ref	
**Hypoglycemia**				
PLHIV	5	11.9	NA	*0.22*
HIV negative	0	0.0	NA	
**Severe anemia**				
PLHIV	6	14.3	3.9 [0.5-7.9]	*0.19*
HIV negative	1	3.9	Ref	
**High parasitemia (> 100000p/µL)**				
PLHIV	1	2.4	0.08 [0.02-0.58]	*0.03*
HIV negative	6	23.1	Ref	
**AST 3XN**				
PLHIV	5	11.9	NA	*0.61*
HIV negative	0	0.0	NA	
**ALT 3XN**				
PLHIV	6	14.3	3.1 [0.58-10.2]	*0.49*
HIV negative	1	3.9	Ref	

*Number of symptomatic individuals in each group: PLHIV (N = 42) and HIV-negative participants (N = 26).*

The incidence of acute renal failure and hypoglycaemia did not differ significantly between the two groups. However, high parasitaemia was significantly more common in HIV-positive patients than in HIV-negative ones (*p = 0.03*). (Table 2).

### Clinical forms of malaria according to HIV status

There was a significant association between the clinical form of malaria and gender, irrespective of HIV status. Men were more likely to be diagnosed with moderate malaria (34.1%; n = 15/44; *p < 0.01*), whereas women were more likely to present with severe malaria (35.9%; n = 28/78; *p < 0.01*). The prevalence of neurological symptoms was also higher in women (47.9%; n = 22/45) than in men (26.1%; n = 6/23), although this difference did not reach statistical significance (*p = 0.07*). The rates of anaemia were 66.7% (n = 44/66) in women and 38.2%; (n = 13/34) among men; and severe anaemia frequency was significantly higher in women (9.1% (n = 6/66) vs 2.9% (n = 1/34); *p = 0.01)*.

The analysis of sociodemographic and clinical-biological features of participants according to malaria severity revealed differences based on HIV status (Table 3). Among individuals aged under 30 years, a higher proportion of HIV-negative participants presented with asymptomatic malaria compared to their HIV-positive counterparts. Conversely, in the age group 30–54 years, the frequency of moderate malaria was significantly higher in HIV-negative individuals compared with PLHIV (Table 3). Among women, HIV-negative participants were predominantly asymptomatic (60.5%; n = 23/38), whereas HIV-positive ones were more likely to present with severe malaria (52.5%; n = 21/40).

**Table 3:** Clinical forms of malaria according to HIV status.

Variables	Asymptomatic Malaria		Moderate Malaria		Severe Malaria		*p*
	PLHIV N = 14	HIV negative N = 40	PLVIH N = 15	HIV negative N = 18	PLHIV N = 27	HIV negative N = 8	
**Age (years), median**	41.1 [32.0-46?0]	43.0 [26,5-52]	38.0 [35-45,5]	35.0 [25.0-48.0]	39.0 [30.0-45,7]	36.0 [26.5-46.0]	
**Age group in years, n (%)**							
< 30 (n = 30)	3 (30.0)	11 (55.0)	2 (20.0)	7 (35.0)	5 (50.0)	2 (10.0)	*0.05*
30-54 (n = 71)	8 (21.1)	21 (63.6)	10 (26.3)	7(21.2)	20 (52.6)	5 (15.2)	<*0.01*
> 54 (n = 21)	3 (37.5)	8 (61.5)	3 (37.5)	4 (30.8)	2 (25.0)	1 (7.7)	*0.44*
**Gender, n (%)**							
Female	10 (25.0)	23 (60.5)	9 (22.5)	9 (23.7)	21 (52.5)	6 (15.8)	< *0.01*
Male	4 (25.0)	17 (60.7)	6 (37.5)	9 (32.1)	6 (37.5)	2 (7.2)	*0.02*
**Temperature, °C [IQR]**			38.0[37-38.5]	39.0 [38-39.5]	38.2[37.4-39]	38.7[38-40]	<0.01
**Anemia, n (%)**	9(64.2)	4(10.0)	11 (73.3)	7(38.9)	22(81.5)	4(50.0)	<0.01
**Median Parasitemia,**							
p/µL**[IQR]**	513 [53-1050]	14 [4-67]	180 [95-600]	3873 [235-53166]	280 [113-957]	100000 [52500-175000]	<0.01
**CTX use, n (%)**	3 (21.4)		7 (46.7)		11 (40.7)		*0.3*
**ARV, n (%)**	12 (100.0)		10 (66,7)*		18 (78.3)*		*0.2*
**CD4 +** cells/mm^3^, **n (%)**							*0.03*
**< 200**	4 (28.6)		10 (76.9)		13 (56.5)		
**200-500**	7 (50.0)		2 (15.4)		10 (43.5)		
**> 500**	3 (21.4)		1 (7.7)		0 (0.0)		

*:N=23

The frequency of anaemia increased with malaria severity in both PLHIV and HIV negatives (*p < 0.01*). The median parasitaemia was significantly higher in cases of asymptomatic malaria co-infection among PLHIV (513 [IQR: 53–1050] p/µL) compared to those with a single malaria infection (14 [IQR: 4–67] p/µL; *p < 0.01*). Conversely, HIV-negative participants with moderate or severe malaria presented with higher parasite densities than PLHIV (Table 3).

Regarding the use of CTX, asymptomatic PLHIV on chemoprophylaxis had higher parasitaemia levels (560 [IQR: 63–978] p/µL) than those not receiving CTX (70 [IQR: 56–1066] p/µL) (*p < 0.01*). Similar trends were observed in the case of moderate malaria (130 [IQR: 69–155] p/µL vs 344 [IQR: 229–675] p/µL; *p < 0.01*) and severe malaria (184 [IQR: 114–958] p/µL vs 308 [IQR: 173–1920] p/µL; *p < 0.01*). There was no significant association between malaria severity and the use of ART or CTX (*p = 0.2*). In addition, among PLHIV, individuals with CD4 counts > 500 cells/µL were more likely to present with asymptomatic malaria, whereas severe malaria was more frequent among immunocompromised patients (CD4^+^ cell count < 200 cells/µL) (*p = 0.03*). The median CD4 + cell count was significantly higher in HIV-positive participants with asymptomatic malaria (304 [IQR: 189–476] cells/µL) compared to those with moderate (180 [IQR: 93–243] cells/µL) or severe malaria (150 [IQR: 90–204] cells/µL) (*p = 0.018*).

### Hb median level during clinical malaria according to HIV status

**Fig 2:**
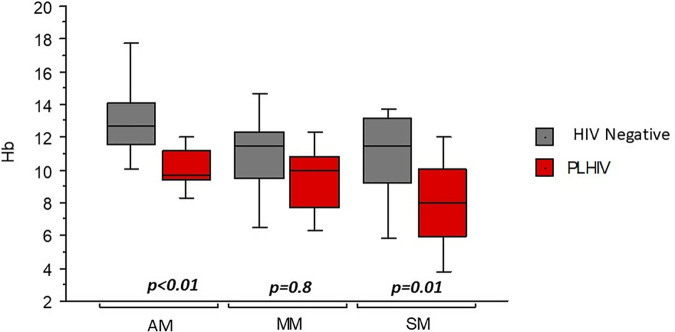
Hb median level according to clinical malaria forms and HIV status. **Legend:** AM: asymptomatic malaria; MM: moderate malaria; SM: severe malaria.

Median Hb levels also differed between HIV-positive and HIV-negative participants (Fig 2). Among those with asymptomatic malaria, the median Hb level was 9.7 [9.4–11.2] g/dL in PLHIV compared to 12.5 [11.6–14.1] g/dL in HIV-uninfected patients (*p < 0.01*). In case of moderate malaria, the median Hb was 10.0 [7.7–10.7] g/dL in PLHIV versus 11.5 [9.5–12.3] g/dL in HIV-negative participants (*p = 0.03*). For severe malaria, PLHIV had a significantly lower Hb level (8.0 [5.9–10.1] g/dL) than HIV-negative patients (11.5 [9.2–13.2] g/dL) (*p < 0.01*). (Fig 2)

## Discussion

Although several studies have documented the risk of severe malaria among PLHIV, data specific to Gabon remain limited. Malaria symptoms are often non-specific, leading clinicians to attribute febrile illness in PLHIV primarily to opportunistic infections, which are the main leading cause of hospital admission in this population [15,23,24]. Our findings highlight that malaria, while sometimes overlooked, can manifest with greater severity in PLHIV, alongside with asymptomatic and moderate forms. By highlighting the spectrum of asymptomatic and complicated form of this disease in a single study, we provide epidemiological insights into the parasite reservoir and clinically actionable information to improve malaria case management among PLHIV in co-endemic settings.

The prevalence of asymptomatic malaria among PLHIV was 6.9%, consistent with previous reports from Libreville, where Bouyou et *al*. (2018) found a frequency of 7.1%, and with 11.9% reported in Ghana by Owusu et *al.* (2017) [13,25]. Among HIV-negative participants, asymptomatic malaria was observed in 8.2%, a rate slightly lower than national estimates of 12.8% and 15.9% recorded respectively in 2019 2022 [18,26]. Prevalence rates in neighbouring countries demonstrate substantial heterogeneity; for instance, Cameroon has reported 59.9%, and the Republic of Congo 48.2% [27,28]. The comparatively lower prevalence observed in this Gabonese PLHIV participants supports a potential protective effect of ART, as previously observed in similar settings [13,14]. The prevalence of complicated malaria among hospitalized PLHIV with febrile illness was 16.1%, closely aligning with the 17.8% reported in Nigeria in 2022, though slightly below the pooled estimate of 27.3% from a recent meta-analysis [29,30]. Thus, despite ART and prophylaxis, clinical malaria remains an important contributor to febrile illness in PLHIV, this reinforces the need for vigilant diagnosis and prompt treatment in endemic settings.

HIV infection is associated with an increased risk of clinical malaria, particularly complicated forms, including severe malaria likely due to HIV-related immunosuppression and malaria-induced immune activation [31,32]. Although comparative studies on the incidence of severe malaria between PLHIV and HIV-negative individuals remain limited, the present results, albeit based on a relatively small sample, highlight the elevated risk of severe malaria among PLHIV. As shown in Table 1, *P. falciparum*-positive PLHIV were more likely to present with neurological symptoms and severe anaemia, consistent with previous reports [33,34]. Neurological manifestations such as prostration (16.7%), repeated seizures (14.3%) and impaired consciousness (26.2%) were more frequent in PLHIV on admission, although these differences did not reach statistical significance. Comparable findings have been reported in Mozambique (14.9% in PLHIV vs 8% in HIV-negative), South Africa (16% vs 8%) and Zambia (56%) [31,34,35]. These neurological symptoms are non-specific and may also result from opportunistic infections such as cryptococcosis and cerebral toxoplasmosis, which are common causes of hospitalization among PLHIV in the IDW-CHUL [36]. Even if malaria was the final diagnosis for the study population, it would be valuable to further assess the burden of neurotropic infectious diseases in PLHIV to better understand the complex clinical presentations associated with malaria-HIV co-infection. Such an approach would improve patient triage and support the implementation of diagnostic and management algorithms that systematically include malaria screening.

In patients with clinical malaria, the median parasitaemia was significantly lower in PLHIV compared to HIV-negative individuals, consistent with observations from Kenya [37]. In a study of asymptomatic co-infected PLHIV in Libreville, the median parasite density was also lower in this group compared with their HIV-negative counterparts [13]. Furthermore, 48.2% of PLHIV had severe malaria, in contrast to only 12.1% of HIV-negative participants. The higher rate of severe malaria in PLHIV supports past findings by Cohen et al. (2022), who reported that HIV co-infection significantly increased 4.15 times the risk of severe malaria [38]. These data underscore the significant role of HIV-related immunosuppression in increasing the risk of severe malaria, emphasising the need for targeted interventions in co-infected individuals.

Anemia was particularly frequent among HIV-malaria co-infected individuals, in line with observations from Mozambique and Ethiopia [39,40]. Its pathogenesis is multifactorial: malaria associated hemolysis of parasitized erythrocytes suppresses erythropoiesis, and increases destruction of non-parasitized red blood cells, while HIV contributes via chronic immune activation and opportunistic infections, affecting hematopoiesis [41,42]. Zidovudine-based ART has been linked to hematologic toxicity, but none of the participants in this study received AZT, as the Gabonese national HIV program had already transitioned to tenofovir-based regimens [43]. Most patients were on TDF-3TC/FTC-EFV, a few initiated DTG-based therapy. The observed hematologic alterations are therefore most likely driven by HIV-malaria co-infection itself. A uniform Hb level threshold was applied for anemia, facilitating comparison with national datasets, though it slightly differs from WHO sex- and age-specific cut-offs. This approach may underestimate mild anemia in women and anemia in men, nevertheless it reflects routine diagnostic practice and ensures consistency with regional studies.

Co-infection may exert additive or synergistic effects, resulting in higher incidence and severity of anemia. Women predominated in our study population. They are generally considered to be at higher risk of anaemia due to menstruation, nutritional deficiencies, and pregnancy-related physiological changes. In line with this, the prevalence of severe anaemia was higher among women than men [44]. However, this pattern also suggests that, among our study participants, other determinants such as HIV status, malaria infection, degree of immunosuppression, and possibly unmeasured factors including inadequate iron intake and parasitic infections, may have played a substantial role in influencing haemoglobin levels beyond sex alone [45].Consistent with other regional reports such as, in Cameroon (56.9%) and Ghana (67%), these findings indicate that anemia remains a major complication in HIV-malaria co-infected patients. This emphasizes the importance of integrated management strategies that address nutritional deficiencies, control parasitemia, and optimize ART adherence to mitigate anemia severity. Even though participants with major opportunistic infections were excluded, unmeasured factors such as intestinal helminth infections or micronutrient deficiencies may have also contributed to anemia. Futhermore, CTX inhibits folate metabolism and could theoretically impair erythropoiesis in individuals with poor folate intake or malabsorption. In this study, CTX use was not associated with increased anemia prevalence, suggesting that its antifolate effect was minimal or compensated by nutritional factors or routine folate supplementation. Similarly, the absence of AZT-containing regimens minimizes the risk of drug-induced marrow suppression. Taken together, anemia in the study participants is most plausibly driven by the combined effects of HIV-related inflammation, malaria-induced hemolysis, immune dysfunction and nutritional deficiencies rather than ART or CTX toxicity. Future studies should incorporate measurements of iron, folate, and vitamin B12 status to disentangle these overlapping mechanisms. These results highlight the need for a comprehensive, integrated approach to managing anemia in HIV-malaria co-infection in endemic settings.

CTX prophylaxis is a key component of HIV care, providing protection against opportunistic and some protozoal infections, including malaria. The higher parasite densities observed among participants receiving CTX prophylaxis may appear counterintuitive, given the drug’s known antifolate activity against *P.falciparum*. However, CTX use was more common among individuals with advanced immunosuppression and lower CD4 counts groups who were at higher risk of elevated parasitaemia. This suggests that the association reflects disease severity rather than a pharmacological paradox, alongside with the low rate of CTX use among PLHIV. Variations in adherence and duration of prophylaxis may also have influenced this observation rather than lack of prescription. In Gabon, CTX is systematically prescribed according to national recommendations but, must be purchased by patients, which contributes to poor adherence and incomplete coverage. The absence of an integrated and differentiated HIV care model requiring specialised consultations for CTX renewal further limits accessibility. These constraints illustrate the systemic challenges faced by PLHIV in maintaining prophylactic regimens, even for treatments recommended by the WHO. Furthermore, there is a need to monitor CTX adherence and immune status when interpreting malaria outcomes among PLHIV.

CD4^+^ cell depletion predisposes individuals to severe malaria, including cerebral malaria and severe anaemia. Both AIDS and malaria are regulated by adaptive and innate immune mechanisms, and the decline in immunity associated with HIV infection increases susceptibility to malaria severity [46]. A low CD4^+^ cell count is well documented as being associated with increased susceptibility to infection [14]. Nearly half of the PLHIV with severe malaria had CD4^+^ cell counts below 200/µL, which aligns with other reports linking low CD4^+^ cell counts to increased vulnerability to malaria infection [30]. This association highlights the importance of monitoring and managing CD4^+^ levels in PLHIV, not only to prevent opportunistic infections, but also to mitigate adverse malaria outcomes.

Despite this study is the first in its kind in Gabon some limitations exists. First, the small sample size of the study population, the low number of confirmed malaria cases was expected, as malaria prevalence in adults in our setting is commonly below 13%, and around 8% among PLHIV. As the IDW-CHUL was the only public inpatient facility dedicated to PLHIV during the study period, our data primarily reflects this specific context, but it inevitably restricts power for rare events. Thus, some findings from subgroup analyses with small sample sizes should be interpreted cautiously and need to be confirmed in larger studies. Nevertheless, this study provides a valuable basis for future multicentric investigations. Second, the predominance of women in the study likely reflects the feminisation of the HIV epidemic in Gabon [23; 36]. A similar female predominance has been reported in the volunteers screened for the PARCAM study [17]. Therefore, the sex distribution in our sample is consistent with the epidemiological reality of HIV in Gabon rather than a recruitment bias. Third, data on the use of malaria prevention interventions were not documented. Information on ITN use among non-pregnant adults is limited, with lower usage rates than among children. National coverage remains below 20%, according to the latest demographic and health survey 2019–2022 [11].

Future research should address co-infections, nutrition, and long-term immune status to better understand malaria-HIV interactions. Standardised syndromic screening complemented by multiplex molecular panels for bacterial, viral, and helminthic pathogens would help quantify the contribution of co-infections to fever, anaemia, and malaria outcomes in PLHIV. Nutritional status should be systematically assessed using harmonised biomarkers ferritin and transferrin saturation with inflammation adjustment, folate, vitamin B12, C-reactive protein, and full blood count, while prospectively documenting iron and folate supplementation. Longitudinal cohorts should capture current and nadir CD4 counts, viral load suppression, ART regimen, and inflammatory markers to understand how immune status influence parasitaemia, anaemia, and disease severity. Study designs should further integrate CTX exposure, vector exposure (ITN use, season, and setting), and parasite antifolate-resistance markers (*pfdhfr/pfdhps*), together with quantitative parasite load (microscopy and qPCR) to understand host, pathogen, and pharmacological effects in high-transmission African settings.

## Conclusion

Severe malaria was more frequent among hospitalised adults living with HIV than among HIV-negative individuals in our study centre, whereas asymptomatic infections predominated among HIV-negative participants. These findings highlight the need to consider HIV status in the clinical management of malaria and support the continued use of integrated preventive strategies such as CYX prophylaxis and INTs. Further larger longitudinal studies are warranted to better characterise the outcomes and neurological impact of malaria-HIV co-infection and to evaluate targeted interventions aimed at improving patient outcomes.

## Abbreviations

HIV: Human immunodeficiency virus
PLHIV: people living with HIV
WHO: World Health Organization
SSA: Sub Saharan Africa
CTX: Cotrimoxazole
IDW-CHUL: Infectious Diseases Ward of Centre Hospitalier Universitaire de Libreville
ART: Antiretroviral treatment
AST: Aspartate aminotransferase
ALT: Alanine aminotransferase
aOR: adjusted Odds Ratio
LFT: Liver function test
CANTAM-EPI 3: Central Africa Clinical Research Network 3
ITN: insecticide treated net
Hb: Haemoglobin
